# Assessing the prevalence of Female Genital Schistosomiasis and comparing the acceptability and performance of health worker-collected and self-collected cervical-vaginal swabs using PCR testing among women in North-Western Tanzania: The ShWAB study

**DOI:** 10.1371/journal.pntd.0011465

**Published:** 2023-07-06

**Authors:** Tamara Ursini, Salvatore Scarso, Stella Mugassa, Jeffer Bhuko Othman, Amina Jumanne Yussuph, Edgar Ndaboine, Gladys Mbwanji, Cristina Mazzi, Martina Leonardi, Marco Prato, Elena Pomari, Humphrey Deogratias Mazigo, Francesca Tamarozzi

**Affiliations:** 1 Department of Infectious-Tropical Diseases and Microbiology, IRCCS Sacro Cuore Don Calabria Hospital, Negrar di Valpolicella, Verona, Italy; 2 School of Public Health, Department of Epidemiology and Behavioural Sciences, Catholic University of Health and Allied Sciences, Mwanza, Tanzania; 3 Department of Medical Parasitology, Catholic University of Health and Allied Sciences, Mwanza, Tanzania; 4 Department of Obstetrics and Gynaecology, Bugando Medical Centre, Mwanza, Tanzania; 5 School of Public Health, Catholic University of Health and Allied Sciences, Mwanza, Tanzania; Federal University of Agriculture Abeokuta, NIGERIA

## Abstract

**Background:**

Female Genital Schistosomiasis (FGS) is a neglected disease of the genital tract due to the inflammatory response to the presence of *Schistosoma haematobium* eggs in the genital tract. The WHO has prioritized the improvement of diagnostics for FGS and previous studies have explored the PCR-based detection of *Schistosoma* DNA on genital specimens, with encouraging results. This study aimed to determine the prevalence of FGS among women living in an endemic district in North-western Tanzania, using PCR on samples collected though cervical-vaginal swabs, and to compare the performance of self-collected and healthcare worker–collected (operator-collected) samples, and the acceptability of the different sampling methods.

**Methods/Principal findings:**

A cross-sectional study was conducted involving 211 women living in 2 villages in the Maswa district of North-western Tanzania. Urine, self-collected and operator-collected cervical-vaginal swabs were obtained from participants. A questionnaire was administered, focusing on the comfortability in undergoing different diagnostic procedures. Prevalence of urinary schistosomiasis, as assessed by eggs in urine, was 8.5% (95%CI 5.1–13.1). DNA was pre-isolated from genital swabs and transported at room temperature to Italy for molecular analysis. Prevalence of active schistosomiasis, urinary schistosomiasis, and FGS were 10.0% (95% CI 6.3–14.8), 8.5% (95%CI 5.1–13.1), and 4.7% (95%CI 2.3–8.5), respectively. When real-time PCR was performed after a pre-amplification step, the prevalence of active schistosomiasis increased to 10.4% (95%CI 6.7–15.4), and FGS to 5.2% (95%CI 2.6–9.1). Of note, more cases were detected by self-collected than operator-collected swabs.

The vast majority of participants (95.3%) declared that they were comfortable/very comfortable about genital self-sampling, which was indicated as the preferred sampling method by 40.3% of participants.

**Conclusions/Significance:**

The results of this study show that genital self-sampling followed by pre-amplified PCR on room temperature-stored DNA is a useful method from both technical and acceptability point of views. This encourages further studies to optimize samples processing, and identify the best operational flow to allow integration of FGS screening into women health programmes, such as HPV screening.

## Author summary

Female Genital Schistosomiasis (FGS) is a severe debilitating disease of the female genital tract, caused by infection with *Schistosoma haematobium*, the agent of urogenital schistosomiasis. It is estimated that FGS affects 20–56 million girls and women, especially in sub-Saharan Africa. The diagnosis of FGS is currently difficult, and this disease often remains undiagnosed or is misdiagnosed as sexually transmitted diseases or genital cancer, with dramatic health and social consequences for the affected women. The WHO has prioritized the improvement of diagnostics for FGS. In this study, we applied and compared the performance of two genital sampling methods (self-collected and healthcare worker (operator)-collected cervical-vaginal swab) followed by molecular analysis, and assessed their comparative acceptability among 211 women living in a *S*. *haematobium* endemic district in North-western Tanzania. We found that not only self-collection by genital swab was well accepted, and actually the preferred sampling method for a high proportion of participants, but that PCR had higher sensitivity when applied to self-collected samples as compared to operator-collected swabs. Furthermore, we used room temperature-stored DNA, which offered greater ease of sample preservation and transportation after extraction. This encourages further studies to optimize samples processing and identify the best operational flow to allow integration of FGS screening into women health programmes, such as HPV screening.

## Introduction

Schistosomiasis is a neglected tropical disease (NTD) caused by infection with trematodes of the genus *Schistosoma*, affecting around 250 million people in tropical and sub-tropical regions, the majority of whom in Africa [1]. *Schistosoma haematobium*, causing urogenital schistosomiasis, is estimated to cause about two thirds of *Schistosoma spp* infections in sub-Saharan Africa [2]. Female Genital Schistosomiasis (FGS), caused by the chronic inflammation elicited by parasite eggs trapped in the female genital tract [3,4] has been estimated to affect 20–56 million girls and women [5,6] and probably represents the most neglected tropical gynaecological condition [3,4]. Chronic lesions are associated with debilitating and stigmatizing morbidity, including vaginal discharge and bleeding, pelvic pain, painful intercourse, infertility, and obstetric problems [5]. Importantly, FGS has been also identified as a cofactor in the acquisition of HIV and probably other sexually transmitted infections (STI) such as Human Papillomavirus (HPV) [3,4,7,8]. Early diagnosis and treatment are paramount to avoid the development of irreversible lesions [1].

The diagnosis of active *S*. *haematobium* infection is routinely based on microscopy of concentrated urine or Polymerase Chain Reaction (PCR) on urine or possibly plasma [9–11]; while CAA circulating antigen detection is not yet widely available [12]. However, the diagnosis of FGS is challenging, and the development of point-of-care diagnostic tests for this disease has been flagged as a priority by the WHO in the 2021–2030 roadmap for NTDs [13]. FGS may occur in the absence of parasite egg excretion in urine [14], and clinical manifestations are largely non-specific, often causing misdiagnosis with STIs or cancer such as cervical cancer, with attendant health and social consequences [15–17]. Parasite eggs are also frequently missed on samples obtained for Pap smear test [18]. The identification of FGS relies on colposcopy inspection, and the visualization of the characteristic lesions [19]. Therefore, availability of equipment and trained gynaecologists are required, representing a significant limitation in the diagnosis of this condition, adding to the discomfort and possible reluctance to attend gynaecological visits [20]. In addition, the colposcopy examination may be normal in a proportion of cases [21]. The reference standard for the diagnosis of FGS, tissue biopsy, has also suboptimal sensitivity and its invasiveness, with related risks and consequences, clearly limits its applicability [4].

Screening for HPV on self-collected genital samples has proven as accurate as diagnosis on operator-collected samples and highly acceptable by participants [22,23]. Therefore, such self-collection strategy is highly appealing for the implementation of screening for FGS, which could be also integrated in HPV screening and/or other female health activities [24,25]. Recent studies have explored the PCR-based detection of *Schistosoma* DNA on genital specimens, including operator-collected cervico-vaginal lavage and self-sampled cervical or vaginal swabs, with encouraging results especially for the use of swabs and the acceptability of self-sampling [14,26,27].

This study aimed to determine the prevalence of FGS among women living in a *S*. *haematobium* endemic district in North-western Tanzania, applying and preliminary comparing the performance of two genital sampling methods (self-collected and operator-collected swab) followed by molecular analysis, and to assess the acceptability of self-collected versus healthcare worker-collected cervical-vaginal sampling.

## Methods

### Ethics statement

Ethical clearance was granted from the Ethics Committee of the provinces of Verona and Rovigo, Italy (protocol N 20607 of 28/03/2022) and from the National Institute for Medical Research (NIMR), Dar es Salaam, Tanzania (NIMR/HQ/R.8a/Vol IX/4019 of 07/06/2022). NIMR also granted authorization for material transfer from Tanzania to Italy for molecular analyses. Clearance from local health authorities was also granted. Identifying data of each participant were recorded in an Informed Consent Form (ICF), coupled with the unique participant ID used to identify all study data and specimens in a pseudonymized manner. The unique participant ID was generated automatically in sticky labels. All ICF documents are stored in a locked cabinet at Catholic University of Health and Allied Sciences (CUHAS), Mwanza, Tanzania, physically separated from all other data collection supports. Only the participant ID code was included on all other paper-based study documents or Excel files. All samples were also labelled with the unique participant ID code by stickers. All analyses were only carried out identifying the participant by the unique ID code.

### Study design and objectives

Cross-sectional study involving consenting women aged 18–45 years, having had prior sexual activity, living in the study villages.

The primary objective was to determine the prevalence of FGS among the target women population living in selected villages of Maswa district, North-western Tanzania, based on the *Schistosoma* PCR positivity on at least one genital swab.

Secondary objectives were i) to evaluate and compare the sensitivity of self-collected versus healthcare worker speculum-aided collected cervical-vaginal samples for the diagnosis of FGS using the composite reference of *Schistosoma* PCR positivity on at least one genital specimen, and ii) to assess the acceptability of different sample collection and diagnostic approaches (genital self-sampling, speculum-aided collected genital sampling conducted by female or hypothetically by male healthcare workers)

### Study area

The study was conducted in two villages of the Maswa district of Simiyu region, namely Bukangilija and Ipililo, purposely selected based on available population prevalence data of urinary schistosomiasis. Bukangilija is situated on the Western side and Ipililo on the Eastern side of Maswa district. This district is characterized by permanent and seasonal fresh water rivers, marshes, swamps, and ponds which create a good living and breeding environment for *Bulinus* snail species (specifically *B*. *nasutus*) which acts as intermediate host for *S*. *haematobium*. Despite repeated rounds of mass drug administration (MDA) with praziquantel (PZQ) in this district, some villages still experience high transmission of *S*. *haematobium*. FGS is a public health concern in the area and one study reported an average prevalence of 5% (ranging from 0–11%) [28]. The last MDA, which targeted school children, was conducted in May 2021 and a recent cross-sectional survey among pre-school and school aged children recorded a district mean prevalence of 12.1% [29]. In the adult population, based on urine reagent dipstick, the overall prevalence of microhaematuria was 9.8% (95%CI 8.1–11.6), with female participants having the highest prevalence, 10.7% (95%CI: 8.5–13.3). Based on the urine filtration technique, overall prevalence of *S*. *haematobium* was 8.1% (89/1101, 95%CI 6.6–9.8) with no significant difference between females and males (8.7% vs 7.3%, respectively) [29].

### Participant inclusion criteria and recruitment

Women were eligible for inclusion in the study if aged 18–45 years, having had prior sexual activity (to allow speculum-aided gynaecological visit and genital sampling), resident in the selected villages of Maswa district, irrespective of complaining of urogenital symptoms, and willing to participate in the study as documented by signing the ICF. Exclusion criteria were menstruation at the time of visit (since this limits visualization of the cervix and correct sampling), known pregnancy, documented treatment with PZQ in the past 6 months. Women over 45 were excluded since pre-menopausal vaginal and cervical characteristics may have made the speculum-aided evaluation of the mucosa more difficult. Women having declared no prior sexual activity were excluded since this does not allow the use of a speculum in the visit. Participant recruitment and sampling was conducted in public buildings and local facilities, as appropriate for each village. Before initiating the recruitment, the study team visited the study areas to conduct planning and sensitizing meetings with district and ward/village/community authorities or leaders. The meeting aimed at describing study procedures, offered treatment, and the importance of providing written informed consent for participation in the study. Village administrations were notified of the project and dates of visits for sampling.

On the study days, participants received a consent form in Kiswahili and a research assistant further explained to each participant the content of the consent form, answered any question, completed the eligibility assessment, and collected written/fingerprint consent from all women willing to participate, in the presence of a witness if the identification of the participant could not be proven by a document. Eligible women finally carried out urine and genital sampling.

All consecutive eligible women accessing the study sites and willing to participate in the study were included until reaching the target sample size.

### Study procedures

**Fig 1:**
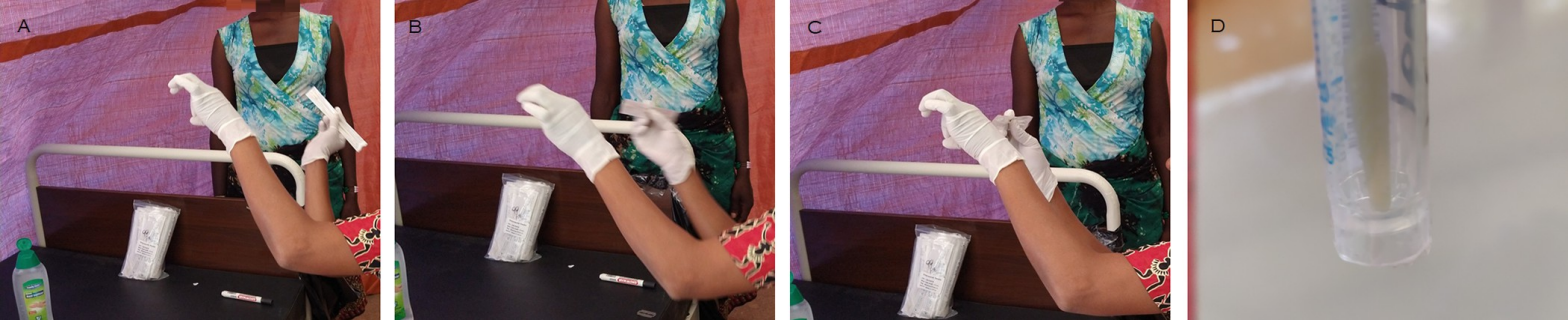
Samples collection. A-C] Explanation of self-sampling. D] Storage of the flocked head of the swab.

Participants were asked to provide one urine specimen, whenever possible collected between 10 am and 2 pm, which was processed for the diagnosis of urinary schistosomiasis using the filtration technique (see “Laboratory analyses”). All genital swabs were collected using CM-FS919 cervical flocked swabs (ShenZhen Cleanmo Technology Co., Ltd) [27]. The participants were instructed on how to self-collect one genital swab [27] (Fig 1), and invited to perform the self-sampling in a private room of the study site. A female nurse was available in case of need, but not formally assisting the participant in the collection, after the explanation. Briefly, each participant was invited to wash hands with water and soap or sanitize them with alcohol-based hand sanitizer. Then, the participant was instructed to position in a comfortable squat position, and gently introduce a flocked swab in the vagina, holding it from the non-flocked end, until some resistance was felt. In that position, the swab had to be gently rotated against the vaginal walls for 10 times. This arbitrary number was applied to standardize as much as possible the self-collection by participants and increase the chances of blindly swabbing against lesions, which were purposively sampled by the health care worker during the following speculum-aided examination. The swab was then extracted, the flocked end of the stick placed in a clean screw-cap tube, and the swab shaft broken to allow closure of the tube’s cap.

The participants were then asked to answer a structured study questionnaire focusing on the knowledge of urinary schistosomiasis, of FGS, and on the comfortability in undergoing different diagnostic procedures.

The participant then underwent a speculum-aided healthcare worker-performed sampling (from now on referred to as “operator-collected swab”) by female physicians, in a private room. The female physicians had similar skills, were the same in both villages, and although operating independently were available for each other support and consultation throughout the study. The participant was examined in the modified lithotomy position. After wearing latex gloves, the physician inspected the vulva for abnormalities (e.g. uterine prolapse) and for abnormal vaginal discharge. They informed the participant about the insertion of the lubricated speculum. The excess mucus was removed with a cotton-head swab. The flocked swab was then inserted into the cervical canal, rotated 3 times, and then swabbed against the vaginal walls while removing the speculum, taking care of swabbing on any FGS-suggestive lesion. Again, this procedure was defined arbitrarily to standardize the sampling.

The swab was then extracted, the flocked end of the stick placed in a clean screw-cap tube, and the swab shaft broken to allow closure of the tube’s cap.

The swab’s heads, collected in tubes labelled with the participants’ ID codes and sampling type (self- or operator-collected), were stored in cool boxes. Any finding observed during the speculum-aided sampling potentially worth of further medical attention was recorded and written in a brief report provided to the participant at the end of the procedure, together with information on how to seek medical advice, outside the study procedures. All women participating to the sampling procedures received treatment with praziquantel tablets, 600 mg (40 mg/kg once) as a standard of care with food provided by the field team.

### Laboratory analyses

Each laboratory analysis was performed blinded from any data obtained during the genital sampling and from results of the other laboratory tests.

Collected urine samples were examined visually for the presence of macrohematuria and using reagent strips for microhematuria (Hemastix, Siemens healthcare Diagnostics GmbH, Germany). Identification and quantification of *S*. *haematobium* eggs followed the method described by the WHO [30]. Briefly, urine samples were vigorously shaken and 10 mL drawn using a plastic syringe pressed through a polycarbonate filter with 20μm pores. All urine filters were microscopically examined for presence of *S*. *haematobium* eggs by trained medical laboratory technicians. All visible *S*. *haematobium* eggs were counted and recorded.

The genital swab specimens were stored and transported in cool boxes, then temporarily stored at 4°C for a maximum of 5 days, until transferred from cool boxes into -40°C freezer for storage in the Catholic University of Health and Allied Sciences (CUHAS) laboratory. Pre-isolation of DNA was performed at CUHAS. After thawing, swabs were suspended in 1.5 mL PBS, vortexed for 10 seconds and left on the bench for another hour. 200μL of sample were pre-treated using a proteinase K heating step and the DNA was isolated using QIAamp spin columns (Qiagen QIAamp DNA Mini Kit cat #51306) according to the manufacturer’s instructions. The DNA was dried and kept bound to the silica membrane of the spin columns and transported at room temperature to the molecular parasitology lab of IRCCS Sacro Cuore Don Calabria Hospital in Italy for the elution, amplification, and detection steps.

First, 200μL of DNA were eluted from each sample. For the detection of *Schistosoma* spp DNA, a real-time PCR was performed according to Obeng et al 2008 [31], with minor modifications. This PCR, using Ssp48F and Ssp124R primers and the double labeled probe Ssp78T, has been extensively validated on its specificity [18,26,31]. DNA amplification and detection were performed with the CFX96 Real Time PCR Detection System (BioRad, laboratories). *S*. *haematobium* DNA positive and negative (NTC, no template control) controls were included in each PCR run and, in addition, a second real-time PCR based on human beta-actin gene was performed on each sample to verify the quality of mucosal sampling and to detect any inhibition of amplification [32]. The reactions were performed using SsoAdvanced Universal Probes Supermix (BioRad). The amplification consisted of 3 min at 95°C followed by 45 cycles of 15 seconds at 95°C, 30 seconds at 60°C, and 30 seconds at 72°C. The output in threshold cycles (Ct) was analysed using BioRad CFX Maestro software. Based on previous reports [33], the positive detection was determined with Ct value ≤ 45, whilst the negativity was determined with no signal of amplification.

In order to improve the detection [34], we also analyzed specifically the self-collected genital samples in a separate experiment introducing a pre-amplification of 20 cycles PCR endpoint using the HotStarTaq Master Mix (Qiagen) and the same primers set Ssp48F and Ssp124R. Briefly, the pre-amplification consisted of 15 min at 95°C followed by 20 cycles of 15 seconds at 94°C, 30 seconds at 60°C and 30 seconds at 72°C, and 5 minutes at 72°C of final extension. The reaction was performed onto C1000 Touch Thermal Cycler (BioRad). Then, 5μL of the PCR product were used as template of the real-time PCR as described above. A confirmatory Dra1 *S*. *haematobium*-specific real-time PCR with pre-amplification step [9] was performed on samples that tested positive on PCR with pre-amplification but negative with the first real-time PCR, to verify the specificity of the result.

### Sample size and data analysis

It has been estimated that up to 75% of women with *S*. *haematobium* infection will also experience FGS [4]. Sensitivity of PCR on any genital sample (self-collected and/or operator-collected) was assumed at 80%, with 100% specificity [27]. We calculated that a sample size of 200 would give a 95% confidence interval with a width of 6%, assuming an expected prevalence of FGS of 5% [28,29].

Results of diagnostic tests were described as binary (pos/neg) as well as using continuous values when appropriate (PCR results as Ct values, number of parasite eggs/10 mL urine). Variables were described as percentage or median with interquartile ranges, as appropriate. Prevalence of FGS was calculated as number of women who test positive for PCR on at least one genital specimen, regardless of whether it was self-collected or collected by the health care worker, on the total population examined. The proportion of FGS among the population with evidence of current infection (i.e., those who test positive for eggs in urine or at least one positive genital swab) was also calculated. Prevalence rates are presented along with their exact 95% confidence intervals (CI).

The agreement between the two genital sample collection methods was performed by Cohen’s Kappa statistics; values ≤ 0 indicated no agreement, 0.01–0.20 none to slight, 0.21–0.40 fair, 0.41–0.60 moderate, 0.61–0.80 substantial, and 0.81–1.00 almost perfect agreement [35]. Binary variables are compared using Chi-square test with simulated p-values; continuous variables are compared using the Wilcoxon-Mann-Whitney test or the Wilcoxon signed rank exact test in the case of paired observations. Correlation between number of eggs per 10 mL urine and PCR Ct values was analysed by Spearman rho correlation coefficient. Relation between Ct of real-time PCR without and with pre-amplification was evaluated by interclass correlation coefficient (ICC). A p-value ≤0.05 was considered statistically significant.

## Results

A total of 211 women participated in the study, 49 from Bukangilija and 162 from Ipililo, between July and August 2022. Raw project data are available online in a data repository. DOI 10.5281/zenodo.7763030

### Urinary and genital schistosomiasis

Urine and self-collected swabs were obtained from all participants, while operator-collected swabs were not performed in 6 participants, because these patients left the study site before undergoing operator-collected swabs. Results are summarized in Table 1.

**Table 1:** Summary of results of urine microscopy and PCR on self-collected and operator-collected genital swabs in the two examined villages.

Laboratory results	Bukangilija	Ipililo	p-value
Eggs in urine positive n/N (%)	11/49 (22.4%)	7/162 (4.3%)	**0.001** ^**§**^
Eggs/10 mL urine median (IQR)^	39 (5–53)	23 (11–36)	0.214*
Microhaematuria whole cohort n/N (%)	18/49 (36.7%)	26/159 (16.4%)	**0.005** ^**§**^
Microhaematuria–active schistosomiasis cohort° n/N (%)	11/13 (84.6%)	7/8 (87.5%)	1.000^**§**^
Self-collected genital swab positive PCR	6/49 (12.2%)	4/162 (2.5%)	**0.014** ^**§**^
PCR Ct on self-collected genital swab median (IQR)^	34.5 (27.7–38.5)	31.0 (26.1–35.9)	0.426*
Operator-collected genital swab positive PCR	5/49 (10.2%)	2/156 (1.3%)	**0.007** ^**§**^
PCR Ct on operator-collected genital swab median (IQR)^	28.6 (23.6–31.5)	34.1 (33.2–35.1)	0.127*

°Active schistosomiasis: positive in at least one assay among urine microscopy, self-collected genital swab, or operator-collected genital swab.

^Calculated within the subgroup of participants positive for the laboratory test

§ Chi-square test.

* Wilcoxon-Mann-Whitney test. Statistically significant differences are indicated in bold.

In 70% of cases, urine was collected between 10 am and 2 pm. Eggs were detected in 18/211 participants (prevalence 8.5%, 95%CI 5.1–13.1), with a significantly higher rate (p = 0.001) in Bukangilija (22.4%, 95% CI: 11.8–36.6) compared to Ipililo (4.3%, 95% CI: 1.8–8.7). Only 2 participants had macroscopic haematuria, therefore all analyses concerning haematuria refer to the results of microhaematuria. In line with the known relationship between haematuria and urinary schistosomiasis [36], microhaematuria was detected in 44 (20.9%) participants, with significantly higher frequency (p = 0.005) in Bukangilija (36.7%) compared to Ipililo (16.4%); agreement between presence of haematuria and of eggs in urine was moderate (Cohen’s Kappa 0.52; 95%CI 0.37–0.67).

In all genital swabs, correct amplification of the human beta-actin gene control target was achieved. At least one genital swab positive for *Schistosoma* by PCR was detected in 10 participants (6/49 [12.2%] in Bukangilija and 4/162 [2.5%] in Ipililo; p = 0.011), representing 4.7% (95%CI 2.3–8.5) overall prevalence of FGS. This figure coincides with prevalence of FGS diagnosed with just self-collected sampling, since, of note, in no case the operator-collected swab was positive and the self-collected swab was negative, while the contrary was true in 3 cases (Table 2). Agreement between PCR on self-collected and on operator-collected swabs in terms of binary result (pos/neg test) indicated almost perfect agreement (Cohen’s Kappa 0.82, 95%CI 0.61–1.00). However, for those cases with both swabs PCR positivity, Ct values were lower in operator-collected compared to self-collected swabs (p = 0.049).

**Table 2:** Comparison of results of *Schistosoma* PCR on self-collected and operator-collected swabs. No Ct: no detected amplification; Ct ≥35: low amount of DNA; ≤30 Ct < 35: moderate amount of DNA; Ct <30: high amount of DNA.

		Operator-collected swab Ct values of *Schistosoma* PCR				
		No Ct	Ct ≥35	≤30 Ct < 35	Ct <30	Total
**Self-collected swab Ct values of *Schistosoma* PCR**	No Ct	195	0	0	0	195
	Ct ≥35	1	0	2	1	4
	≤30 Ct < 35	1	0	0	1	2
	Ct <30	1	1	1	1	4
Total		198	1	3	3	205

When accounting for at least one test positive for active schistosomiasis (eggs in urine and/or at least one positive genital swab), prevalence of active *S*. *haematobium* infection was 10.0% (95% CI 6.3–14.8), 26.5% in Bukangilija (95% CI: 14.9–41.1) and 4.9% in Ipililo (95% CI: 2.2–9.5). No statistically significant differences were found between the two villages for what concerned parasite egg output and PCR Ct from self-collected and operator-collected genital swabs (Table 1).

**Fig 2:**
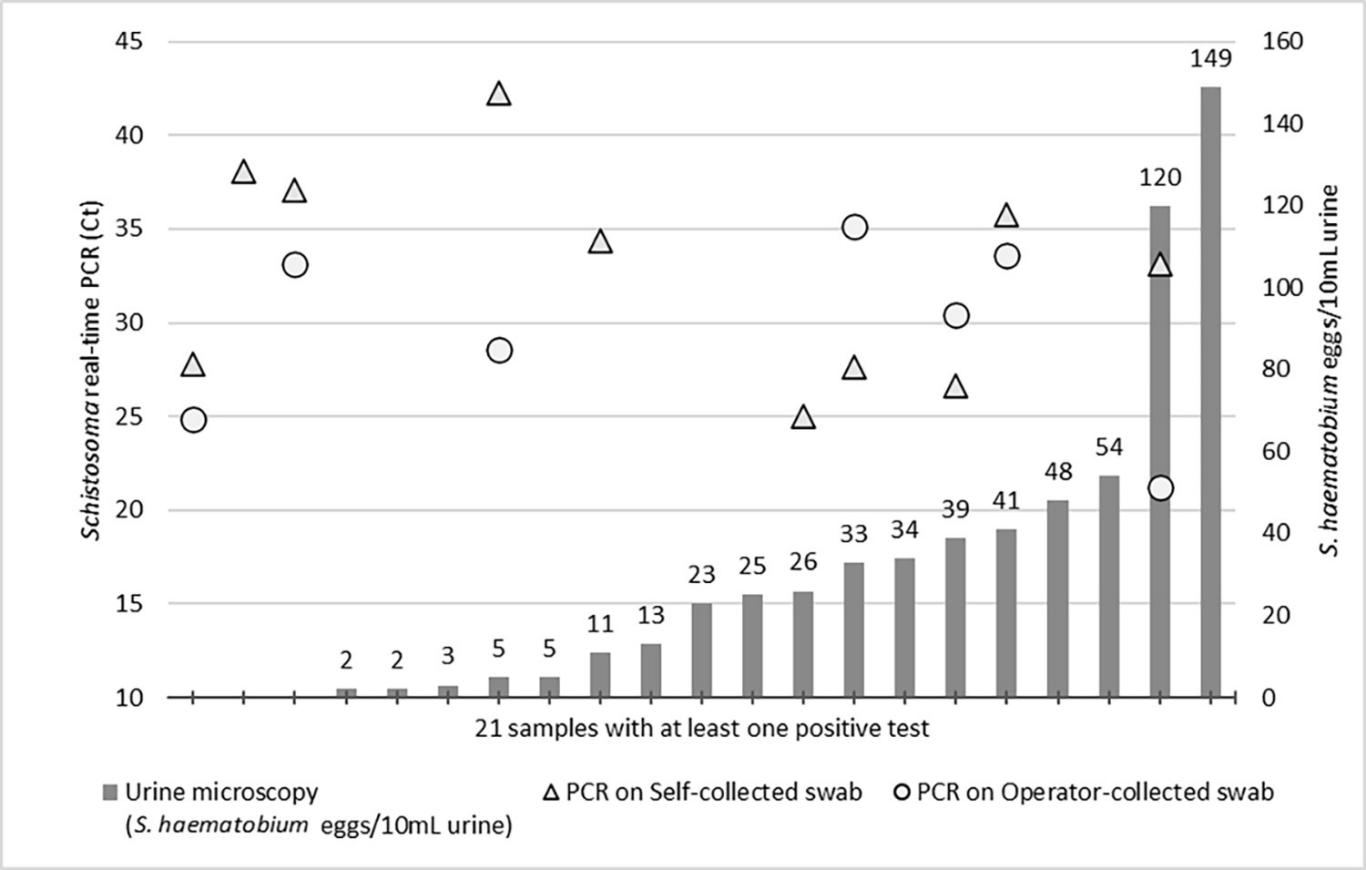
Active *S*. *haematobium* infections based on positivity to at least one test among urine microscopy, real-time PCR from self-collected swab and real-time PCR from operator-collected swab. On x-axis are represented participants with active schistosomiasis; the numbers above bars represent the number of eggs/10 mL urine as indicated on the right y-axis. Triangles and dots indicate positivity on self-collected and operator-collected swab, respectively, at Ct values indicated on the left y-axis.

There was only moderate agreement between presence of FGS, as assessed by positivity in at least one genital swab, and presence of *Schistosoma* eggs in urine (Cohen’s Kappa 0.47; 95%CI 0.23–0.70). Also, no correlation was found between number of eggs per 10 mL urine and PCR Ct values of self-collected (Spearman rho = 0.145; p = 0.567) or operator-collected swabs (Spearman rho 0.276; p = 0.268) (Fig 2).

Similarly, no agreement was found between FGS and presence of haematuria (Cohen’s Kappa 0.19; 95%CI 0.05–0.34).

### Pre-amplification of DNA from self-collected genital swabs

When self-collected genital samples were analyzed by PCR following a first pre-amplification step, all samples that tested positive on the first real-time PCR were also found positive, as expected, at lower Ct values. Intraclass correlation of PCR Ct results without and with pre-amplification was 0.799 (95% CI 0.743–0.843). In addition, one further sample was detected as positive, which was negative on the first real-time PCR. The positivity was confirmed using the Dra1 *S*. *haematobium*-specific real-time PCR with pre-amplification, on both self-collected and operator-collected swabs. Thus, the prevalence of FGS and of active urogenital schistosomiasis could be re-calculated as 5.2% (95% CI 2.6–9.1) and 10.4% (95%CI 6.7–15.4), respectively.

This sample came from a participant living in Bukangilija who had no eggs in urine, no haematuria, and yellow cervical patches on speculum-aided examination.

### Vaginal examination

Vaginal examination was aided by a disposable transparent speculum; colposcopy could not be performed due to unavailability of portable equipment. Vaginal discharge was observed in 26 (12.3%), and vaginal bleeding in 15 (7.1%) participants. One or more vaginal and/or cervical lesions were observed in 20 participants (9.5%). Lesions were described as cervicitis (n = 5), Nabothian cysts (n = 6), yellow/sandy patches (n = 2), rubbery papules (n = 1), hyperaemic/erythematous lesions (n = 3), and abnormal vascularization (n = 2); in 1 case the description was missing. Of note, only two women with FGS as assessed by positive PCR from genital swab presented abnormalities at the speculum-aided examination: in both cases vaginal discharge.

### Study questionnaire

**Fig 3:**
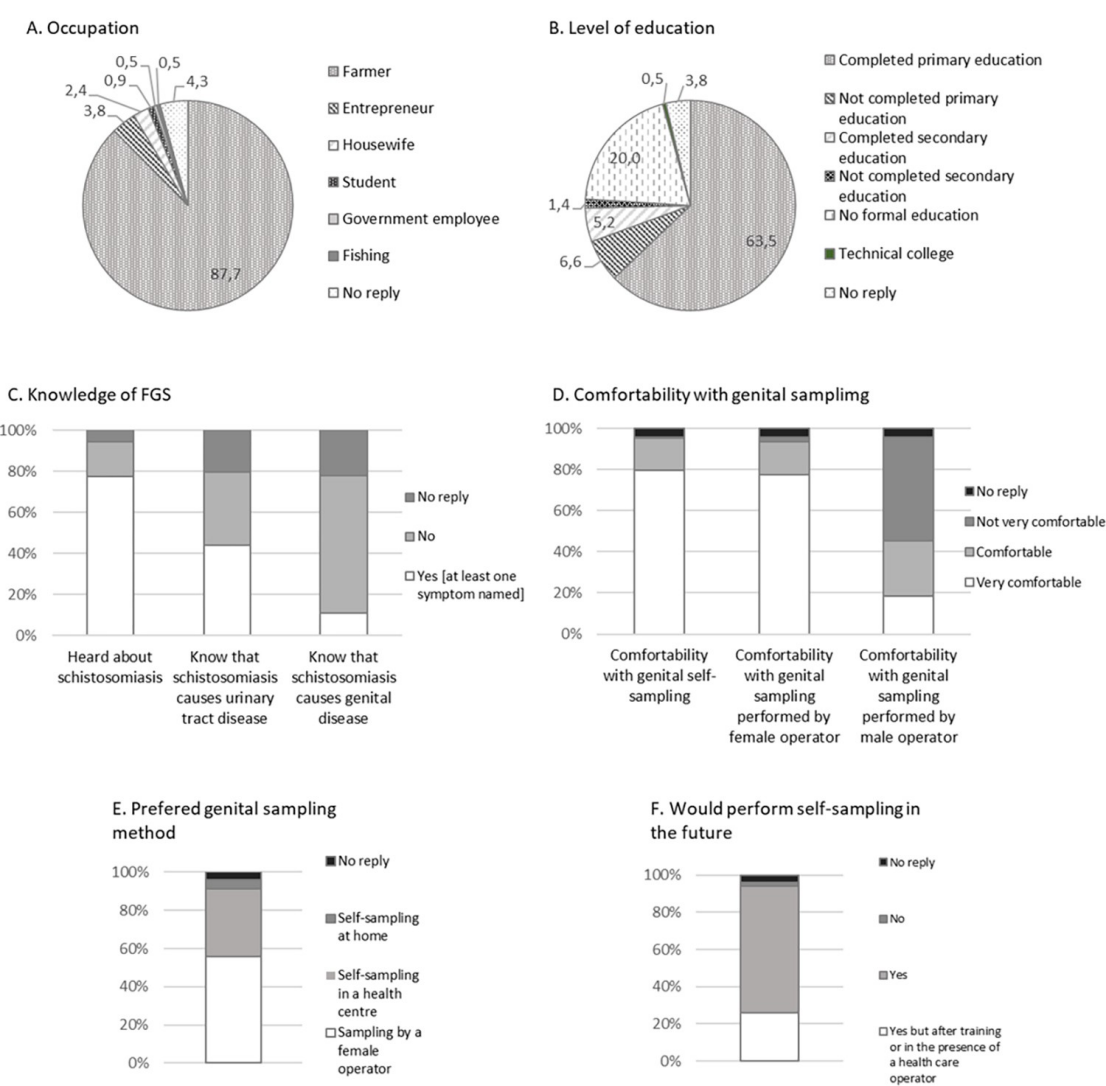
Results of study questionnaire for the 211 participants.

The overall results of the study questionnaire are summarized in Fig 3 and detailed in S1 Table as for what concerns the two villages separately, between which there was no difference. The majority of participating women were farmers (87.7%), had completed primary education (63.5%), and had heard about schistosomiasis (77.3%), but less than a half (44.1%) knew that it caused urinary tract disease, and a minority (10.9%) that it caused genital tract pathology. Furthermore, of those who declared to know about genital schistosomiasis, only about half (53%) could name at least one related symptom.

When participants were requested about their attitudes and preferences towards genital sampling, the majority (95.3%) declared that they were very comfortable or comfortable about genital self-sampling. Also, the majority (93.4%) of participants declared they were comfortable or very comfortable with genital sampling performed by a female health care worker while less than half (45.5%) participants felt comfortable or very comfortable with the perspective of sampling by a male health care worker. Overall, when asked about the preferred genital sampling method, self-sampling (40.3%) and female health care worker sampling (55.9%) were the two preferred modalities. However, there was a clear preference for the health care setting as the place for sampling, since less than 5% participants would prefer to self-sample at home. The majority (94.3%) would perform self-sampling in the future, but about one in four of these would do it after further training or in the presence of a health care worker. Of the minority (4 women) who declared they would not repeat self-sampling in the future, two simply specified that “they could not” and two that they felt they could not be better than an operator in taking samples. Interestingly, though, 3 of these 4 participants had replied to the former question that their preferred method would be actually self-sampling.

## Discussion

In this study, we applied and compared the performance of two genital sampling methods (self-collected and operator-collected cervical-vaginal swab) followed by molecular analysis to determine the prevalence of FGS, and assessed their acceptability, among women living in a *S*. *haematobium* endemic district in North-western Tanzania.

Accurate diagnosis of gynecological pathologies, including FGS, are paramount to apply the appropriate treatment and avoid the development of complications; improved and accessible diagnostics for FGS has been flagged as a priority by the WHO [13]. While FGS in younger girls may benefit from school-based Mass Drug Administration with praziquantel, teenagers and adult women are not targeted by these control programmes, but could be reached through women health programmes, such as HPV screening. The availability of easy to apply screening tools, ideally for multiple pathogens, would be paramount for integrated woman health programmes.

With tools currently available, the diagnosis of FGS is difficult [4]. Urine microscopy for *S*. *haematobium* eggs can be negative in FGS [14], clinical manifestations are not specific, and its diagnosis based on evaluation of genital lesions requires equipment and training to recognize specific lesions’ characteristics [37,38]. These conditions are confirmed by the results of this study as well, showing limited agreement between FGS and urinary microscopy, hematuria (used as a proxy for urinary schistosomiasis [39]), and genital lesions observed on a simple speculum-aided gynecological visit.

Prevalence of active schistosomiasis, urinary schistosomiasis, and FGS in the examined population were 10.0% (95% CI 6.3–14.8), 8.5% (95%CI 5.1–13.1), and 4.7% (95%CI 2.3–8.5), respectively. When considering the PCR results obtained on self-sampled swabs after the pre-amplification step the prevalence of active schistosomiasis could be recalculated as 10.4% (95%CI 6.7–15.4), and that of FGS at 5.2% (95%CI 2.6–9.1).

These results are in line with expected figures (on which sample size was calculated), which supports the robustness of the results obtained with our method. Importantly, PCR from self-sampled swabs actually detected more FGS cases compared to operator-collected swabs, supporting the use of this easy-to-collect and accepted sampling technique. Furthermore, the application of a pre-amplification step to the PCR allowed increasing the sensitivity of diagnosis. Of note, and different from what applied in previous similar studies, the DNA extracted was stored for twenty-three days in the extraction columns at room temperature, allowing transportation without maintaining a freezing chain, helping significantly the logistics of samples analysis. Further activities now should focus on the evaluation of the performance of different swab types, of storage of the swabs at temperature conditions minimizing the need for a cold chain, and on the evaluation of the possibility for molecular testing HPV and possibly other genital pathogens on the same swab. This would greatly facilitate, both technically and logistically, the diagnosis of FGS in endemic areas but also in migrant patients attending health care in non-endemic countries, where awareness and diagnostic capacity for neglected tropical diseases is often available only in a few specialized centers. From a broader point of view, sexual and reproductive health services would greatly benefit from including FGS diagnosis, also considering the increased risk of both HIV and HPV transmission associated with FGS [40,41].

The results of the preference and comfortability questionnaire also support the use of genital self-sampling, since almost all participants answered that they would be happy to repeat this procedure in the future. This result is in line with what also found in Zambia [20] and supports the use of this technique in integrated women health programmes. The larger preference expressed for genital sampling from a female healthcare operator, followed by that for self-sampling in a health care center, as opposed to at home, most likely reflects the fact that women in resource-poor areas are happy to take these diagnostic opportunities as a chance to be attended by a physician or nurse also on other health matters of concern. Moreover, in resource-poor conditions, self-sampling at home would probably have much higher logistical complexities than in case of home self-sampling in a high-resource setting.

This study had several limitations, including the limited sample size, the relatively low prevalence of *S*. *haematobium* infection in the area accessible for this investigation, and the unavailability of colposcopy as a reference for the clinical diagnosis of FGS. Also, the diagnosis of active schistosomiasis did not include CAA antigen testing, which could have given a more precise figure of *S*. *haematobium* infection on which to evaluate the prevalence of FGS. Furthermore, we could not interview women who did not attend the study procedures, to know what barriers prevented them from participating. This clearly biases to some degree the results of the preference and comfortability questionnaire, which was answered only by women who *a-priori* had some positive attitude towards the applied procedures, and should be the object of further operational investigations.

To conclude, we applied genital swab sampling followed by molecular analysis for the diagnosis of FGS and compared the results obtained from two sampling methodologies. Our results show that genital self-sampling followed by PCR is a useful method from both technical and acceptability point of views. Further studies, ideally in multiple and different settings in terms of *S*. *haematobium* prevalence and clinical context, should be performed to optimize samples processing, especially storage, and identify the best operational flow in each setting.

## Supporting information

S1 Table Overall results of the study questionnaire. (DOCX)
