# Supplementary material for: Assessing the prevalence of Female Genital Schistosomiasis and comparing the acceptability and performance of health worker-collected and self-collected cervical-vaginal swabs using PCR testing among women in North-Western Tanzania: The ShWAB study
Source: PLoS Negl Trop Dis. 2023 Jul 6;17(7):e0011465. doi: 10.1371/journal.pntd.0011465 (PMC10353784; doi:10.1371/journal.pntd.0011465)
Supplement: S1 Table — (DOCX) [file pntd.0011465.s001.docx]

# **Supporting information**

**S1 Table. Overall results of the study questionnaire**

| **Question** | **Bukangilija (n=49) N (%)** | **Ipililo (n=162) N (%)** | **Total (n=211) N (%)** |
| --- | --- | --- | --- |
| **Occupation** | | | |
| Farmer | 47 (95.9%) | 138 (85.2%) | 185 (87.7%) |
| Entrepreneur | 0 | 8 (4.9%) | 8 (3.8%) |
| Housewife | 0 | 5 (3.1%) | 5 (2.4%) |
| Student | 1 (2.0%) | 1 (0.6%) | 2 (0.9%) |
| Government employee | 0 | 1 (0.6%) | 1 (0.5%) |
| Fishing | 1 (2.0%) | 0 | 1 (0.5%) |
| No reply | 0 | 9 (5.6%) | 9 (4.3%) |
| **Level of education** | | | |
| Completed primary education | 25 (51.0%) | 109 (67.3%) | 134 (63.5%) |
| Not completed primary education | 4 (8.2%) | 10 (6.2%) | 14 (6.6%) |
| Completed secondary education | 7 (14.3%) | 4 (2.7%) | 11 (5.2%) |
| Not completed secondary education | 1 (2.0%) | 2 (0.8%) | 3 (1.4%) |
| No formal education | 12 (24.5%) | 28 (17.2%) | 40 (19.9%) |
| Technical college | 0 | 1 (0.6%) | 1 (0.5%) |
| No reply | 0 | 8 (4.9%) | 8 (3.8%) |
| **Heard about schistosomiasis** | | | |
| Yes | 42 (85.7%) | 121 (74.7%) | 163 (77.3%) |
| No | 5 (10.2%) | 31 (19.1%) | 36 (17.1%) |
| No reply | 2 (4.1%) | 10 (6.2%) | 12 (5.7%) |
| **Know that schistosomiasis causes urinary tract disease** | | | |
| Yes | 21 (42.9%) | 72 (44.4%) | 93 (44.1%) |
| No | 25 (51.0%) | 50 (30.9%) | 75 (35.5%) |
| No reply | 3 (6.1%) | 40 (24.7%) | 43 (20.4%) |
| **Know that schistosomiasis causes genital disease** | | | |
| Yes [at least one symptom named] | 8 (16.3%) [5 (62.5%)] | 15 (9.3%) [8 (53.3%)] | 23 (10.9%) [13 (56.5%)] |
| No | 38 (77.6%) | 103 (63.6%) | 141 (66.8%) |
| No reply | 3 (6.1%) | 44 (27.2%) | 47 (22.3%) |
| **Comfortability with genital self-sampling** | | | |
| Very comfortable | 42 (85.7%) | 126 (77.8%) | 168 (79.6%) |
| Comfortable | 6 (12.2%) | 27 (16.7%) | 33 (15.6%) |
| Not very comfortable | 1 (2.0%) | 0 | 1 (0.5%) |
| No reply | 0 | 9 (5.6%) | 9 (4.3%) |
| **Comfortability with genital sampling performed by female operator** | | | |
| Very comfortable | 45 (91.8%) | 118 (72.8%) | 163 (77.3%) |
| Comfortable | 3 (6.1%) | 31 (19.1%) | 34 (16.1%) |
| Not very comfortable | 1 (2.0%) | 4 (2.7%) | 5 (2.4%) |
| No reply | 0 | 9 (5.6%) | 9 (4.3%) |
| **Comfortability with genital sampling performed by male operator** | | | |
| Very comfortable | 9 (18.4%) | 30 (18.5%) | 39 (18.5%) |
| Comfortable | 18 (36.7%) | 39 (24.1%) | 57 (27.0%) |
| Not very comfortable | 22 (44.9%) | 84 (51.9%) | 106 (50.2%) |
| No reply | 0 | 9 (5.6%) | 9 (4.3%) |
| **Preferred genital sampling method** | | | |
| Sampling by a female operator | 28 (57.1%) | 90 (55.6%) | 118 (55.9%) |
| Self-sampling in a health centre | 13 (26.5%) | 62 (38.3%) | 75 (35.5%) |
| Self-sampling at home | 8 (16.3%) | 2 (0.8%) | 10 (4.7%) |
| No reply | 0 | 8 (4.9%) | 8 (3.8%) |
| **Would perform self-sampling in the future** | | | |
| Yes | 35 (71.4%) | 109 (67.3%) | 144 (68.2%) |
| Yes but after training or in the presence of a health care operator | 11 (22.4%) | 44 (27.2%) | 55 (26.1%) |
| No | 3 (6.1%) | 1 (0.6%) | 4 (1.9%) |
| No reply | 0 | 8 (4.9%) | 8 (3.8%) |
